# Social determinants and behavioural factors influencing toothbrushing frequency among primary school children in rural Australian community of Lithgow, New South Wales

**DOI:** 10.1186/s13104-020-05239-3

**Published:** 2020-08-28

**Authors:** Amit Arora, Subrat Nargundkar, Paul Fahey, Hema Joshua, James Rufus John

**Affiliations:** 1 School of Health Sciences, Western Sydney University, 24.2.97 Campbelltown Campus, Locked Bag 1797, Penrith, NSW 2751 Australia; 2 Translational Health Research Institute, Western Sydney University, Locked Bag 1797, Penrith, NSW 2751 Australia; 3 Discipline of Child and Adolescent Health, Sydney Medical School, Faculty of Medicine and Health, The University of Sydney, Westmead, NSW 2145 Australia; 4 Oral Health Services, Sydney Local Health District and Sydney Dental Hospital, NSW Health, Surry Hills, NSW 2010 Australia; 5 Rozetta Institute, Rozetta Institute (Formerly Capital Markets Cooperative Research Centre), The Rocks, Sydney, NSW Australia

**Keywords:** Toothbrushing, Socioeconomic status, Children, Rural, Non-fluoridated, Oral health

## Abstract

**Objective:**

This study aims to determine the social determinants and behavioural factors influencing frequency of toothbrushing among primary school children residing in the rural community of Lithgow in New South Wales, Australia. All six primary schools of Lithgow were approached to participate in a cross-sectional survey prior to implementation of water fluoridation. A validated oral health survey questionnaire was completed by 703 parents of the children. Multivariable logistic regression analysis was employed to determine significant predictors associated with frequency of toothbrushing.

**Results:**

Parents with a positive attitude towards water fluoridation had 74% higher odds (OR = 1.74, 95% CI 1.17–2.60) of their children brushing twice or more daily. Children living in a single parent household had 34% reduced odds (OR = 0.66, 95% CI 0.43–0.99) of brushing twice daily. Poor maternal oral health was significantly associated with suboptimal dental hygiene practices in children, where mothers who had any tooth extracted had 7% reduced odds of their children brushing their teeth twice or more daily (OR = 0.93, 95% CI 0.90–0.97). Subsequently, children with increased consumption of chocolates per day were less likely to brush twice or more daily. Finally, children with dental insurance had two times higher odds (OR = 2.04, 95% CI 1.40–2.96) of brushing twice daily.

## Introduction

Dental caries is recognised as a global public health concern [1]. Amongst 354 diseases considered in the Global Burden of Disease study (1990–2017), untreated dental caries was the most ubiquitous disease [2, 3]. In Australia, dental caries is the most common health issue in childhood [4]. The recent Australian National Child Oral Health Survey (NCOHS) 2012–2014 reported that over 25% of 5 to 10-year-old children had untreated caries in the primary dentition, while one in ten children aged 6 to 14 years had untreated caries in the permanent dentition [5].

Although dental caries has imminent negative health consequences, it is largely preventable by regular toothbrushing with a fluoridated toothpaste along with other measures such as a low sugary diet, regular dental visits, and water fluoridation [6]. Toothbrushing using a fluoridated toothpaste is one of the most effective methods to prevent dental caries [7] and the motor skills required for toothbrushing are developed from an early age to adolescence [8]. The fluoride in the toothpaste promotes enamel remineralisation through the formation of fluorapatite crystals [9]. A Cochrane review reported that parental supervision and children’s frequency of toothbrushing enhances the protective effect of fluoridated toothpaste [10]. However, the most recent Australian NCOHS reported that only 50% of children aged 5 to 14 years brushed their teeth twice daily with a fluoridated toothpaste [5].

There is ample evidence on health inequality as a result of geographical remoteness, limited fluoride exposure, access to dental services, and affordability [11]. The Australian national survey reported that children living in rural and remote areas had 38% higher proportion of untreated caries than those living in major cities [5] due to a multitude of factors such as lack of water fluoridation, socio-economic status, and shortage of dental workforce [12].

Lithgow Local Government Area (LGA) within the jurisdiction of former Sydney West Area Health Service (now under Nepean Blue Mountains Local Health District) is a recently fluoridated community in NSW [13–15]. Studies have been conducted to ascertain dental caries burden in Lithgow children prior to water fluoridation [13, 14]. Although there is some evidence on the oral health of rural Australian children, there is paucity of evidence on the predictors of toothbrushing frequency among rural children. Therefore, the aim of this study is to identify the factors influencing toothbrushing frequency among primary school children in the rural Lithgow community, Australia.

## Main text

### Study background

This study is a secondary data analysis of the cross-sectional survey on primary school children in rural non-fluoridated community of Lithgow, prior to water fluoridation in 2014 [13, 15]. All six primary school principals in Lithgow LGA gave permission to conduct the survey. The parents of children were then invited to take part in a survey via a take-home information pack. Further details of our previous work are mentioned elsewhere [13–15].

### Data collection

For standardised collection of information, the dental survey questionnaire was adapted from the Australian NCOHS as used in our previous studies [13, 14]. The socio-demographic information collected in the questionnaire include child-specific characteristics including child’s age, gender, age when toothbrushing commenced, discretionary diet, and dental visit history. Additionally, family or parental characteristics include parent’s age, education, occupation, country of birth, marital status, extraction history, language spoken at home, private health insurance (PHI) status, and family income. The primary outcome of this study was a dichotomised version of toothbrushing frequency: brush at least twice per day (coded yes or no). Clinical dental examination of children’s oral health was performed by primary researchers. The guidelines of the World Health Organization (WHO) were adopted as the diagnostic criteria for dental caries [16].

### Statistical analysis

A theoretical model based on previous literature with use of Fisher-Owen’s framework [17] was employed wherein all variables present in the model were fitted in the multiple logistic regression analyses to determine the factors that were independent predictors of toothbrushing frequency in Lithgow LGA community. All variables were tested against the outcome variable and were adjusted for other covariates in the multivariable regression analysis where a backward stepwise method was used to eliminate variables that had a non-significant effect in a stepwise manner. All variables in the final model were variables for which, when excluded, the change in deviance compared with the corresponding Chi-square (*Χ*^2^) test statistic on the relevant degrees of freedom was significant (p < 0.05).

In addition, variables were tested for collinearity using Pearson’s product-moment collinearity tests against each other and against other covariates, before including them in multivariable logistic regression analysis. However, all variables tested had correlations of less than 0.5 implying that the possibility of collinearity between the variables is small.

One sample z-tests of proportions were performed to compare the data collected from this survey with Australian Bureau of Statistics census of Lithgow region for 2011 [18] for help determining the external validity of the data. All statistical analyses were undertaken using the IBM SPSS Statistics version 24.

## Results

Of the 1400 parents contacted in the Lithgow LGA, 703 (52.1%) completed the survey questionnaire. The descriptive statistics shows that only 65% of children brushed twice or more daily whereas 35% of children brushed once or less daily (Table 1).

**Table 1:** Socio-behavioural factors influencing tooth-brushing frequency in primary school children of LGA (n = 703)

Socio-behavioural factors	n^a^	Tooth-brushing Frequency		Chi square^b^	p value
		< 2/ day (n = 247)	≥ 2/ day (n = 454)		
Child-specific characteristics					
Age of the child, mean (SD)	703	8.7 (2.0)	8.9 (2.0)	0.004^c^	0.324
Gender of the child					
Female	348	120 (48.6)	228 (50.2)	0.172	0.679
Male	353	127 (51.4)	226 (49.8)		
Age when toothbrushing commenced					
Less than 12 months of age	45	12 (5.1)	33 (7.8)	1.742	0.187
12 months or more	612	223 (94.9)	389 (92.2)		
Last visit to dentist					
Less than 12 months	536	178 (72.4%)	358 (79.0%)	3.968	0.046
12 months or more	163	68 (27.6%)	95 (21.0%)		
Serves of sugar sweetened beverages per day					
0	78	23 (9.3)	55 (12.1)	25.499	< 0.001
1	132	28 (11.3)	104 (22.9)		
2	171	54 (21.9)	117 (25.8)		
3	138	59 (23.9)	79 (17.4)		
4 or more	182	83 (33.6)	99 (21.8)		
Serves of chocolate per day					
0	235	64 (25.9)	171 (37.7)	15.600	< 0.001
1	329	118 (47.8)	211 (46.5)		
2 or more	137	65 (26.3)	72 (15.9)		
Family-specific characteristics					
Marital status of parents					
Married or having partner	560	177 (71.7%)	383 (84.4%)	16.061	< 0.001
Single parent	141	70 (28.3%)	71 (15.6%)		
Age of mother					
20–29 years	73	36 (14.8%)	37 (8.2%)	9.850	0.007
30–39 years	400	143 (58.6%)	257 (57.0%)		
≥ 40 years	222	65 (26.6%)	157 (34.8%)		
Age of father					
20–29 years	25	10 (5.7)	15 (3.9)		
30–39 years	275	94 (54.0)	181 (47.5)		
≥ 40 years	255	70 (40.2)	185 (48.6)		
Education status of mother					
University	170	44 (18.2%)	126 (28.3%)	8.643	0.003
Vocational or High school	517	198 (81.8%)	319 (71.7%)		
Education status of Father					
University	98	22 (12.3%)	76 (20.1%)	5.060	0.024
Vocational or high school	460	157 (87.7%)	303 (79.9%)		
Job of Mother					
Managers and professionals	147	36 (14.8%)	111 (24.9%)	23.694	< 0.001
Skilled workers	309	98 (40.2%)	211 (47.4%)		
Pensioners and employed	233	110 (45.1%)	123 (27.6%)		
Job of father					
Managers and professionals	166	55 (31.6)	111 (29.2)	0.397	0.820
Skilled workers	348	106 (60.9)	242 (63.7)		
Pensioners and employed	40	13 (7.5)	27 (7.1)		
Parental attitude towards water fluoridation					
Negative or unsure	154	72 (29.1)	82 (18.4)	10.657	0.001
Positive	539	175 (70.9)	364 (81.6)		
Extractions due to tooth decay in mother					
No extractions	336	94 (38.1%)	242 (53.3%)	14.901	< 0.001
One or more	365	153 (61.9%)	212 (46.7%)		
Extractions due to tooth decay in father					
No extractions	260	69 (38.8%)	191 (49.5%)	5.632	0.018
One or more	304	109 (61.2%)	195 (50.5%)		
Private dental insurance					
No	405	177 (75.3%)	228 (53.3%)	31.024	< 0.001
Yes	258	58 (24.7%)	200 (46.7%)		
Family income					
More than $100 K	74	20 (10.8%)	54 (16.0%)	21.871	< 0.001
$40–100 K	247	69 (37.1%)	178 (52.7%)		
Up to $40 K	203	97 (52.2%)	106 (31.4%)		

^a^Sample size includes only responding individuals

^b^Pearson chi square test

^c^Unpaired t-test

Table 2 shows unadjusted and adjusted odds ratios of the regression analyses respectively. In the multivariable analysis, positive parental attitude towards water fluoridation and private dental insurance were significantly associated with increased frequency of toothbrushing. Parents with a positive attitude towards water fluoridation had 74% higher odds (OR = 1.74, 95% CI 1.17–2.60) of their children brushing twice or more daily. Children who were covered by a private dental insurance had two times higher odds (OR = 2.04, 95% CI 1.40–2.96) of brushing twice or more daily.

**Table 2:** Univariate and multivariate logistic regression analysis of Tooth-brushing Frequency with non-imputed and imputed models

Socio-behavioural factors	Tooth-brushing frequency			
	Univariable analysis			Multivariable analysis
	Unadjusted odds ratio (95% CI)	p value	Adjusted odds ratio (95% CI)	p value
Age of the child, mean (SD)	1.04	0.323		
Gender of the child				
Female	1.00			
Male	0.94 (0.69, 1.28)	0.679		
Age when tooth brushing commenced			NS	
Less than 12 months of age	1.00			
12 months or more	0.63 (0.32, 1.25)	0.190		
Last visit to Dentist			NS	
Less than 12 months	1.00			
12 months or more	0.69 (0.48, 0.99)	0.047		
Serves of sugar sweetened beverages per day			NS	
0	1.00			
1	1.55 (0.82, 2.95)	0.178		
2	0.91 (0.50, 1.62)	0.741		
3	0.56 (0.31, 1.01)	0.055		
4 or more	0.50 (0.28, 0.88)	0.016		
Serves of chocolate per day				
0	1.00		1.00	
1	0.67 (0.46, 0.96)	0.031	0.60 (0.40, 0.90)	0.013
2 or more	0.41 (0.27, 0.64)	< 0.001	0.41 (0.25, 0.65)	< 0.001
Marital status of parents				
Married or having partner	1.00		1.00	
Single parent	0.46 (0.32, 0.68)	< 0.001	0.66 (0.43, 0.99)	0.044
Age of Mother			NS	
20–29 years	1.00			
30–39 years	1.74 (1.05, 2.89)	0.029		
≥ 40 years	2.35 (1.36, 4.04)	0.002		
Age of Father			NS	
20–29 years	1.00			
30–39 years	1.29 (0.55,2.98)	0.551		
≥ 40 years	1.75 (0.75, 4.08)	0.194		
Education status of mother			NS	
University	1.00			
Vocational degree or high school	0.56 (0.38, 0.82)	0.004		
Education status of father			NS	
University	1.00			
Vocational degree or high school	0.55 (0.33, 0.93)	0.026		
Job of mother			NS	
Managers and professionals	1.00			
Skilled workers	0.69 (0.44, 1.09)	0.114		
Pensioners and unemployed	0.36 (0.23, 0.57)	< 0.001		
Job of father				
Managers and professionals	1.00			
Skilled workers	1.13 (0.76, 1.68)	0.541		
Pensioners and employed	1.03 (0.49, 2.14)	0.939		
Parental attitude towards fluoridation				
Negative or unsure	1.00		1.00	
Positive	1.83 (1.27, 2.63)	0.001	1.74 (1.17, 2.60)	0.007
Extractions due to tooth decay in Mother				
No extractions	1.00		1.00	
One or more	0.53 (0.39, 0.73)	< 0.001	0.93 (0.90, 0.97)	< 0.001
Extractions due to tooth decay in Father			NS	
No extractions	1.00			
One or more	0.64 (0.45, 0.92)	0.018		
Private dental insurance				
No	1.00		1.00	
Yes	2.67 (1.88, 3.80)	< 0.001	2.04 (1.40, 2.96)	< 0.001
Income of the family			NS	
More than $100 K	1.00			
$40–100 K	0.95 (0.53, 1.71)	0.878		
Up to $40 K	0.40 (0.22, 0.72)	0.002		

Independent variables adjusted in the risk model are: Marital status of parents, Age of mother, Education status of Mother, Education status of Father, Job of Mother, Extractions due to tooth decay in Mother, Extractions due to tooth decay in Father, Attitude towards Water Fluoridation, Private dental insurance, Income of the family

*CI* Confidence interval, *NS* Not significant

Model 1—Original (non-imputed data)

Model 2—Imputed data

However, factors such as single parent household, one or more tooth extraction history in mothers, and increased serves of chocolates consumed per day were determined to be significantly associated with decreased frequency of toothbrushing in children. Children living in a single parent household had 34% reduced odds (OR = 0.66, 95% CI 0.43–0.99) of brushing twice daily compared to those living with married parents. Poor maternal oral health was significantly associated with suboptimal dental hygiene practices in children, where mothers who had any tooth extracted had 7% reduced odds of their children brushing their teeth twice or more daily (OR = 0.93, 95% CI 0.90–0.97). Subsequently, children with increased consumption of chocolates per day were less likely to brush twice or more daily.

Table 3 shows the comparison of the socio-demographic characteristics of the Lithgow study population with that of the 2011 Australian Census. It is seen that the expected population estimates of the Lithgow survey did not significantly differ from the Census for factors such as Indigenous status and highest education level in the household. However, it is observed that the children with two Australian born parents were 4% over-represented in the Lithgow survey compared to Census report.

**Table 3:** Population benchmark comparison of demographic characteristics of Lithgow from ABS census 2011 report

Socio-demographic characteristics	Survey estimate (observed percentages) % of children (95% CI)	Observed p-value	2011 census report (expected percentages) % of children
Country of birth of household^a^		< 0.001*	
Overseas	12.02 (9.60–14.42)		16.45
Australia	87.9 (85.36–90.19)		83.55
Indigenous status of household^b^		< 0.001*	
Indigenous	4.42 (2.90–5.94)		5.57
Non-indigenous	95.58 (94.06–97.10)		94.43
Highest education level in the household^c^		0.268	
University or college degree	28.86 (25.50–32.21)		26.83
High school or vocational training	71.14 (67.79–74.50)		73.17

* Statistically significant at 5% level

^a^Children were classified to the overseas born category if they had at least one parent who was born overseas

^b^Children were classified to the Indigenous category if they had at least one parent who was Indigenous

^c^Children were classified to the University or College degree category if they had at least one parent who had a university or college degree

## Discussion

This study provided insights on various factors influencing toothbrushing frequency in primary school children in rural non-fluoridated Lithgow LGA. Approximately 65% of the parents who completed the survey, reported that their children brushed their teeth twice or more daily. This is less than the 75% reported by the AIHW report in 2012 which could be due to remoteness of the area compared to the overall Australian rates [12].

The multivariable analysis show that parents with a positive attitude towards water fluoridation had higher odds of having their children brush twice daily or more with fluoridated toothpaste compared to parents who are unsure or antipathic towards water fluoridation. The positive attitude of parents may be due to the result of increased awareness of the benefits of water fluoridation to oral health, thereby encouraging their children to brushing frequently with a fluoridated toothpaste, as reported in other studies [19].

Children who were covered by a private dental insurance had higher odds of brushing their teeth frequently compared to those who were not covered by a private insurance. The Australian government currently provides Medicare and Pharmaceutical Benefit Schemes to fund the general health expenditure [20]. It is worthy to note that children covered by private health insurance readily have access to comprehensive dental treatment whereas children who are not covered by a private insurance are limited to public dental services, which often have long waiting periods [21]. The descriptive findings of this study show that only 37% of children reported having private insurance which is consistent with other study findings reporting that the lack of private insurance is further exacerbated by residence in rural and remote areas [22]. In addition, disparities in private insurance coverage and optimal oral health care are also evident by socioeconomic status as reported in other studies. Children who are covered by private dental insurance are more likely to come from a family with higher socioeconomic status and therefore have better access and increased visits to the dentist resulting in better dental hygiene practices and experience as opposed to children without private dental insurance [22].

Studies report that the development of oral hygiene practices in children are primarily influenced by the mother’s oral hygiene attitudes and beliefs [23–26]. The study findings show a significantly lower odds of toothbrushing in children whose mothers have poor oral health. Mothers who are less concerned about their oral hygiene and those who under-estimate the importance of oral health eventually would not take notice of their child’s dental hygiene and maintenance, which would lead to negative oral health outcomes. This may be manifested in terms of their children’s discretionary diet, where children having increased serves of chocolates consumed per day also had reduced odds of toothbrushing twice or more daily. This relationship of maternal oral health and toothbrushing frequency of their children is comparable to other studies [24–26].

The study also identified that children in single parent household had reduced odds of brushing their teeth twice or more daily. It has been suggested that lack of paternal support and financial strain causes increased stress may lead to development of suboptimal oral hygiene practices [27, 28]. Emotional stress related to family structure and changing thereof can also contribute to suboptimal oral hygiene practices [28].

Although this study had a response rate of 52%, it does not necessarily lead to bias. In order to help clarify the external validity, comparison with the 2011 census was performed which confirmed that the population estimates of the survey did not differ significantly from the census estimates for indigenous status and education level of household as reported in previous study [29]. However, this survey over-represented the percentage of children born to two Australian born parents by 4% (Table 3) [29].

## Limitations

This study has some limitations. The cross-sectional method used to collect data is limited in establishing temporality between the social determinants and the tooth-brushing frequency [30]. In addition, the self-reported method used in the form of questionnaire allows potential for self-reporting bias [31]. Although the study included a several social determinants and behavioural factors to test against the outcome of toothbrushing frequency, some behavioural factors such as parent’s TB behaviour which were not recorded in the study. In terms of generalisability of the research to the source population, children born to Australian parents were 4 percent over-represented in the survey in comparison to the Census. However, this study provided useful insights on the social determinants and behavioural factors that significantly predict frequent toothbrushing in Lithgow children which could prove valuable in informing oral health promotion and policy development programs. Future scope of research would prove useful to further explore the impact of other possible predictors such as perception of parents on the level of prioritisation of oral health for their children and the role of teachers on oral health promotion in children.

## Data Availability

The data of this study can’t be shared publicly due to the presence of sensitive (confidential) participants’ information.

## Abbreviations

AIHW: Australian Institute of Health and Welfare
CI: Confidence interval
LGA: Local Government Area
NSW: New South Wales
NCOHS: National Child Oral Health Survey
OR: Odds ratio
WHO: World Health Organization
